# Small open reading frames: a comparative genetics approach to validation

**DOI:** 10.1186/s12864-023-09311-7

**Published:** 2023-05-01

**Authors:** Niyati Jain, Felix Richter, Ivan Adzhubei, Andrew J. Sharp, Bruce D. Gelb

**Affiliations:** 1 Department of Genetics and Genomic Sciences and Mindich Child Health and Development Institute, Icahn School of Medicine at Mount, Hess Center for Science and Medicine, 1470 Madison Avenue, New York, NY 10029 USA; 2 Present Address: Committee On Genetics, Genomics, and Systems Biology, The University of Chicago, Chicago, IL USA; 3 Department of Pediatrics, Icahn School of Medicine at Mount Sinai, New York, NY USA; 4 Department of Biomedical Informatics, Harvard Medical School, Boston, MA USA; 5 Division of Genetics, Brigham and Women’s Hospital, Boston, MA USA

**Keywords:** Micropeptides, Small open reading frames, Human genetic variation, Evolutionary conservation, Comparative genetics

## Abstract

Open reading frames (ORFs) with fewer than 100 codons are generally not annotated in genomes, although *bona fide* genes of that size are known. Newer biochemical studies have suggested that thousands of small protein-coding ORFs (smORFs) may exist in the human genome, but the true number and the biological significance of the micropeptides they encode remain uncertain. Here, we used a comparative genomics approach to identify high-confidence smORFs that are likely protein-coding. We identified 3,326 high-confidence smORFs using constraint within human populations and evolutionary conservation as additional lines of evidence. Next, we validated that, as a group, our high-confidence smORFs are conserved at the amino-acid level rather than merely residing in highly conserved non-coding regions. Finally, we found that high-confidence smORFs are enriched among disease-associated variants from GWAS. Overall, our results highlight that smORF-encoded peptides likely have important functional roles in human disease.

**Supplementary Information:**

The online version contains supplementary material available at 10.1186/s12864-023-09311-7.

## Introduction

Open reading frames (ORFs) are stretches of DNA bounded by a stop codon, and represent an important genomic feature used to discover protein-coding genes. Traditionally, ORFs with less than 100 codons, also known as small ORFs (smORFs), were excluded from gene annotations. This arbitrary cutoff was imposed by the challenges of distinguishing protein-coding smORFs from random noise, both experimentally and computationally. However, recent advances in next-generation sequencing and proteomics have identified many smORF-encoded proteins, termed micropeptides [1–3].

smORFs reside in various regions of the genome, including 5’ and 3’ untranslated regions (UTRs), intergenic regions, and even in regions previously annotated as non-coding RNAs (ncRNAs) [1, 4]. Studies have also identified diverse biological functions for a few human micropeptides. For example, mitoregulin (MTLN), a 56-amino-acid (aa) micropeptide, is highly expressed in skeletal and cardiac muscle cells and is involved in regulating mitochondrial complex assembly [5]. Additionally, phospholamban (52 aa), sarcolipin (31 aa), and myoregulin (46 aa) are well-characterized regulators of the cardiac sarco-/endoplasmic reticulum calcium ATPase, SERCA [6]. Notably, mutations in the *PLN* gene are implicated in human dilated cardiomyopathy and heart failure, underscoring its crucial role in cardiac function [7, 8]. As a result, there is growing interest in annotating and functionally characterizing smORFs.

Recent studies combining RNA-sequencing (RNA-seq) and ribosome profiling (Ribo-seq) have identified and annotated thousands of putative smORFs across different human cell lines [1, 4]. While this approach is a highly significant advance over merely identifying ORFs in the human genome, the fraction of those thousands of putative smORFs that are truly protein-coding remains unclear and the functions of the micropeptides they encode remain unknown. To begin to address those uncertainties, functional genomic studies have been performed using mass spectrometry and CRISPR-based mutagenesis screens. These CRISPR-based mutagenesis screens have identified hundreds of smORFs in cell growth phenotypes [4, 9]. Nonetheless, there is still a need to develop methods to validate protein-coding smORFs, since new smORFs are constantly being identified in humans and other species. Therefore, an alternative strategy for identifying *bona fide* smORFs, which has not been attempted previously, is to use the power of comparative genetics, both across human populations and species. We hypothesized that smORFs that are truly protein-encoding should behave similarly to known genes and biologically validated smORFs with respect to human genetic variation and evolutionary conservation.

To examine the patterns of human genetic variation in putative smORFs, we annotated previously published smORF datasets with variants from 71,702 whole-genome sequencing samples from the Genome Aggregation Database (gnomAD) and defined constraint in smORFs using the robust missense observed/expected upper bound fraction (MOEUF) score [10]. Additionally, we characterized smORFs under evolutionary constraint using Genome Evolutionary Rate Profiling (GERP) scores [11]. Together these analyses allowed us to curate a list of high-confidence smORFs. Finally, we examined the role of smORFs in human disease and showed that disease-associated variants identified in genome-wide association studies (GWAS) are enriched in our set of high-confidence smORFs.

## Results

### Overview of smORF datasets

**Fig. 1:**
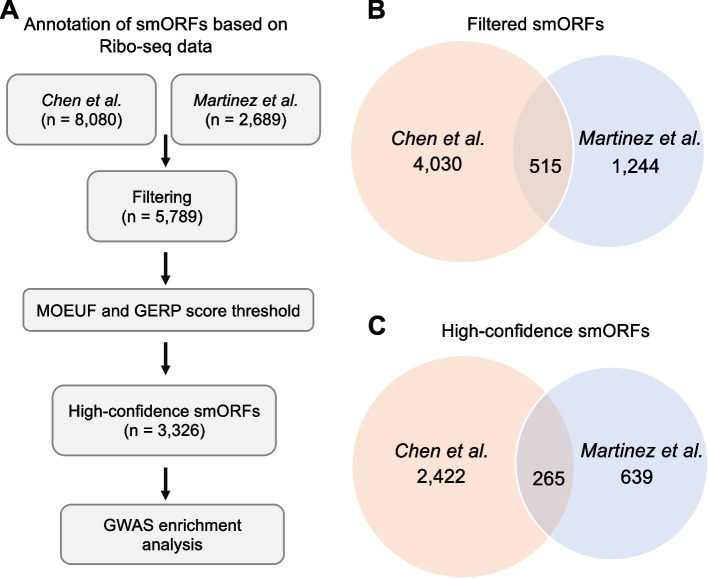
Overview of smORF datasets. **A** Study workflow. **B** Venn diagram showing the overlap between the predictions from Chen et al*.* and Martinez et al*.* study in the filtered smORFs dataset. **C **Venn diagram showing the overlap between the predictions from Chen et al*.* and Martinez et al*.* in the high-confidence smORFs dataset. Abbreviations: MOEUF, missense observed/expected upper bound fraction; GERP, Genome Evolutionary Rate Profiling; GWAS, genome-wide association studies

We analyzed datasets from two recent studies that performed Ribo-seq to annotate ORFs across various human cell lines (Fig. 1A). From each dataset, we first obtained a set of high quality smORFs that were not isoforms of known genes. Chen et al*.* identified 15,236 putative smORFs, and we proceeded with 8,080 smORFs that were not isoforms of known genes [4]. Similarly, Martinez et al*.* reported a gold standard set of 2,689 smORFs that were also not isoforms of known genes [1]. After obtaining putative smORFs, we first filtered them based on amino acid length (range 10–150 amino acids) and selected the longest isoform per smORF. While studies have demonstrated that smORFs that overlap protein-coding genes out-of-frame, are translated independently and can encode functional proteins [12–15], in our analysis we decided to exclude such ORFs as it would be challenging to distinguish those that represent protein-coding smORFs versus annotated genes translated out-of-frame. Specifically, we excluded smORFs overlapping annotated exons in the RefSeq database [16] as well as those overlapping the Peptide Atlas database [17]. In the set of smORFs that passed our initial filtering criteria (*n* = 5,789), we observed 515 smORFs with exact matches that were reported by both studies (Fig. 1B) and an additional 739 smORFs that had imperfect overlap. This minimal overlap may be due to differences in cell types, Ribo-Seq protocols, and ORF annotation tools between the studies. Given the minimal overlap between the two datasets, we considered the entire set of filtered smORFs in our downstream analysis to identify high-confidence smORFs.

### Selection of high-confidence smORFs

**Fig. 2:**
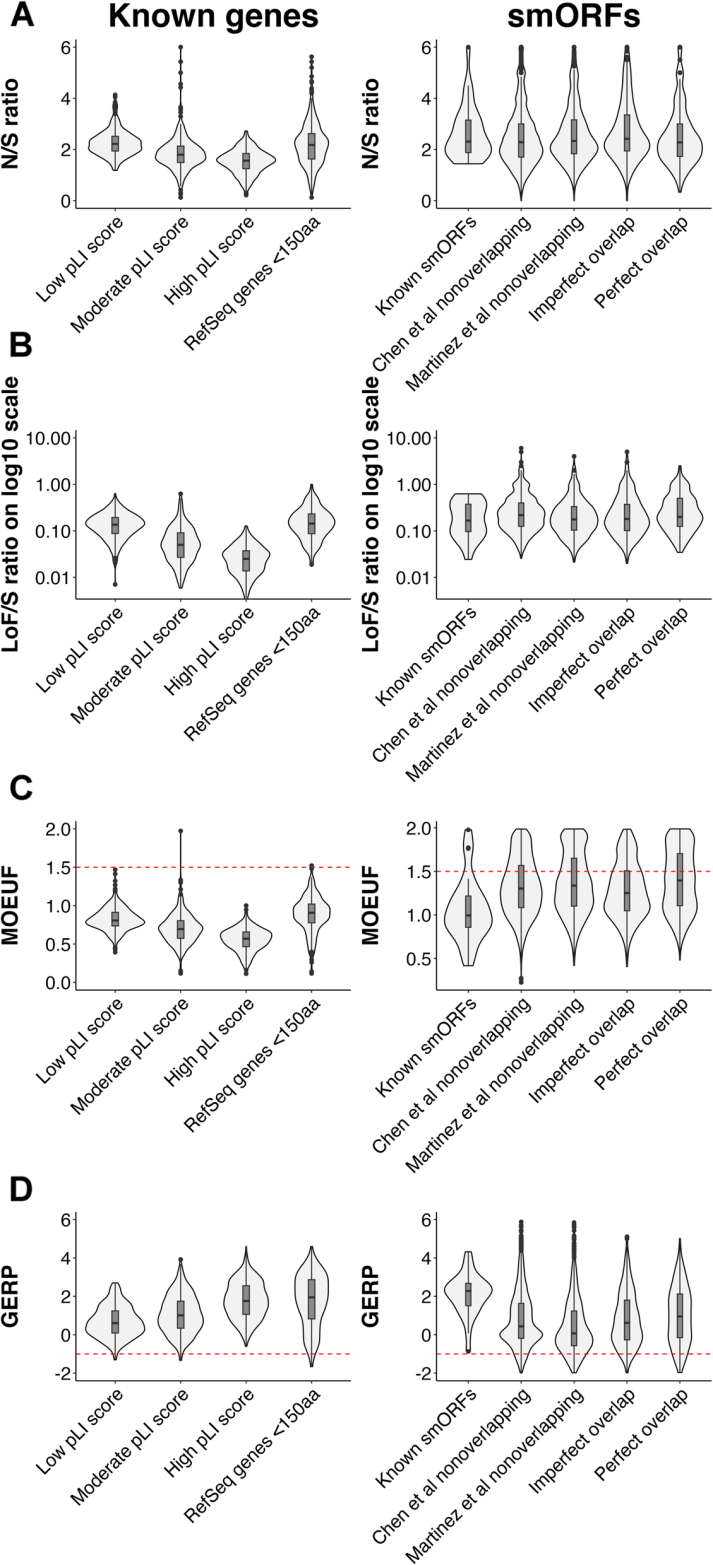
Selection of high-confidence smORFs. For selected RefSeq genes with varying pLI scores and amino acid length < 150 (left facets) and smORFs (right facets), violin and boxplots showing **A** N/S ratios, **B** LoF/S ratios, **C** MOEUF scores, and **D** GERP scores. MOEUF and GERP thresholds used to filter putative smORFs are shown by dashed red line. Selected RefSeq genes are segregated by pLI scores ranging from low (*n* = 400), moderate (*n* = 400) and high (*n* = 400) scores, and genes with less than 150 amino acids (*n* = 400). smORF subsets includes known smORFs (*n* = 28), putative smORFs unique to Chen et al. (*n* = 4,030) and Martinez et al. (*n* = 1,244) dataset, putative smORFs with exact matches reported by both Chen et al. and Martinez et al. (*n* = 515), and smORFs in both datasets with imperfect overlap (*n* = 739). Box plots display the first quartile, median and third quartile. Abbreviations: N, nonsynonymous; S, synonymous; LoF, loss-of-function; pLI, probability of loss-of-function intolerant

Given the limited functional evidence for the smORF predictions, we combined human genetic variation and evolutionary conservation metrics to identify high-confidence smORFs. First, we annotated the filtered list of putative smORFs with human genetic variants from the gnomAD v3 database (*N* = 71,702 genomes) and benchmarked these against a set of known smORFs with extensive biological validation [5, 6, 18–31] (*n* = 28, Table S1), a set of RefSeq genes with amino acid length < 150 and a selection of RefSeq genes [16] with varying probability of loss-of-function intolerant (pLI) scores. pLI is a metric to define genes that are intolerant to loss-of-function (LoF) variants, with scores ranging from 0 to 1 for least to most intolerant [32]. A high pLI score (≥ 0.9) indicates the gene is under strong selective constraint. Consistent with the range of pLI scores in the selected RefSeq genes, we observed a clear pattern in the distribution of non-synonymous/synonymous (N/S) and loss-of function/synonymous (LoF/S) ratios, whereby the median ratios were significantly lower in genes with high pLI scores compared to genes with low pLI scores, as expected (Fig. 2A and B, right facets, Kruskal–Wallis P_N/S_ < 2.2 × 10^–16^ and P_LoF/S_ < 2.2 × 10^–16^). Next, we compared N/S and LoF/S ratios among the different sets of known and putative smORFs. Here, we observed significant differences in the distribution of N/S and LoF/S ratios across the subsets of smORFs (Fig. 2A and B, left facets, Kruskal–Wallis P_N/S_ = 3.23 x 10^-5^ and P_LoF/S_ = 0.00197). Notably, the N/S ratios of the smORF subsets were comparable to RefSeq genes with low pLI scores and higher than RefSeq genes with moderate and high pLI scores (Fig. 2A).

We observed that the number of LoF variants per smORF was usually < 10, which is too few to yield robust loss-of-function OEUF scores [10]. Therefore, to examine constraint within putative smORFs, we instead utilized the comparable missense variation metric, MOEUF, as this could be calculated based on a much larger number of single nucleotide variants (SNVs). MOEUF is the conservative estimate of the upper bound of the confidence interval for the observed-to-expected missense variants ratio. Low MOEUF scores indicate relatively higher intolerance to missense variation, and high MOEUF scores indicate a relatively higher tolerance to missense variation or limited statistical power to detect constraint [10]. While MOEUF examines the constraint of peptide sequences in the context of human populations, evolutionary conservation metrics, such as GERP, identify constrained DNA sequences across multiple species. GERP is based on a multiple mammalian species alignment, with positive scores indicating evolutionary constraint [11]. Therefore, we used MOEUF and GERP scores to provide additional evidence of the biological significance in smORFs.

As expected, the pattern of pLI scores in RefSeq genes was consistent with the degree of constraint based on MOEUF and GERP scores (Fig. 2C and D). Furthermore, known smORFs had MOEUF and GERP scores within the same range as RefSeq genes and were comparable to RefSeq genes with amino acid length < 150, in line with the previous biological functional characterization of these smORFs. Therefore, we utilized the range of MOEUF and GERP scores observed in these biologically validated smORFs and RefSeq genes to set thresholds to further filter predicted smORFs. We selected smORFs with MOEUF scores ≤ 1.5 and GERP scores ≥ -1 as this combination of filters captured almost the entire set of known smORFs (25/28), RefSeq genes with amino acid length < 150 (389/400) and RefSeq genes (1192/1200) while filtering out a considerable proportion of the putative smORFs (Fig. 2C and D, red dashed line). Post filtering, we were left with 3,326 smORFs (31% of all putative smORFs) as our high-confidence set (Table S1). In this high-confidence set, we observed 265 smORFs that were reported by both the Chen et al*.* and Martinez et al*.* (Fig. 1C).

### High-confidence smORFs conserved at the protein-coding level

**Fig. 3:**
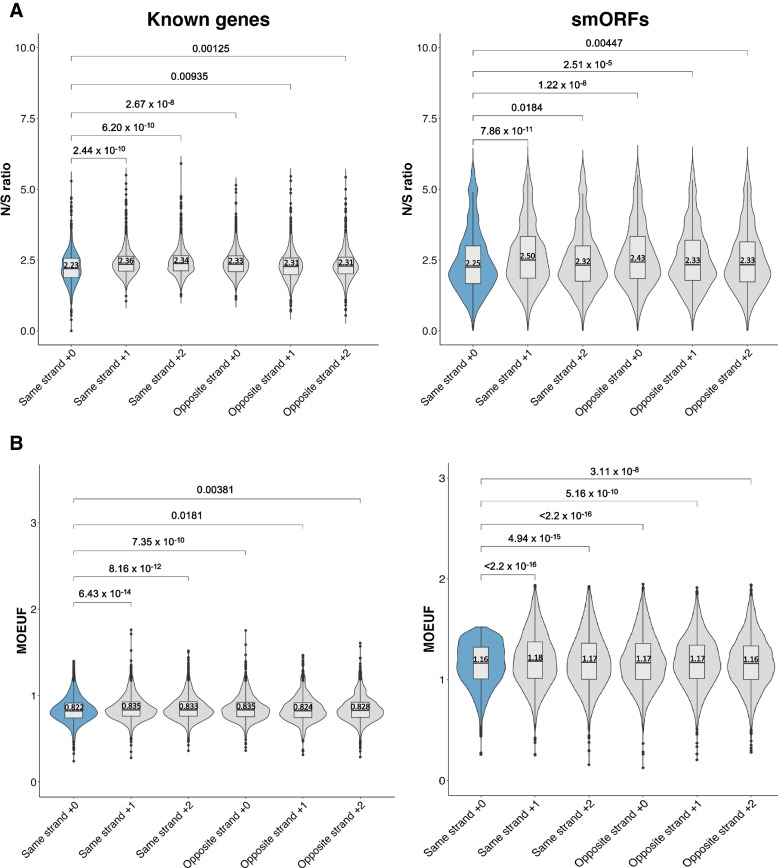
Conservation of high-confidence smORFs at the protein-coding level. **A** Violin and box plots showing the distribution of N/S ratios of the correct (blue) and “incorrect” (grey) reading frames of RefSeq genes (*n* = 1200) and high-confidence smORFs (*n* = 2,891), with a lower N/S ratio observed in the correct reading. **B** Violin plot and boxplot showing that the correct reading frame (blue) had lower MOEUF scores compared to all five “incorrect” reading frames (grey) for both RefSeq genes (*n* = 1200) and high-confidence smORFs (*n* = 2,891). Box plots displaying the first quartile, median and third quartile. All *P* values are based on the paired Wilcoxon signed rank test

While our previous analyses allowed us to filter away putative smORFs of lower confidence, they did not address whether the sequences of the high-confidence smORFs were constrained due to the need to maintain the sequence for the encoded micropeptide per se versus merely residing in a highly constrained genomic region. To address this, we next asked whether the remaining set of filtered smORFs showed evidence of conservation at the amino acid level relative to the genome-wide background rate. We extracted the genomic coordinates for the five “incorrect” reading frames, two on the same strand and three on the opposite strand, for the set of high-confidence smORFs and a set of RefSeq genes. We then compared the N/S ratios and MOEUF scores of the five “incorrect” reading frames to the correct reading frame, providing five negative controls for every smORF and RefSeq gene. As expected, the correct reading frame for RefSeq genes showed significantly lower median N/S ratios (paired Wilcoxon signed rank test, all *P* values < 0.01) and MOEUF scores (paired Wilcoxon signed rank test, all *P* values < 0.02) compared to the other five reading frames. Similarly, we observed that high-confidence smORFs’ correct reading frame had significantly lower median N/S ratios and MOEUF scores than the “incorrect” reading frames (paired Wilcoxon signed rank test, all *P* < 0.02. The N/S ratios and MOEUF scores reflect stronger conservation in RefSeq genes (median Gene_N/S_ = 2.23, Gene_MOEUF_ = 0.822) compared to smORFs (median smORF_N/S_ = 2.25, smORF_MOEUF_ = 1.16), possibly driven by a fraction of smORFs within our high-confidence set that are not true protein-coding genes or by limited statistical power of MOEUF and N/S ratio to detect conserved smORFs (Fig. 3A and B).

### Enrichment of disease-associated GWAS variants in high-confidence smORFs

**Fig. 4:**
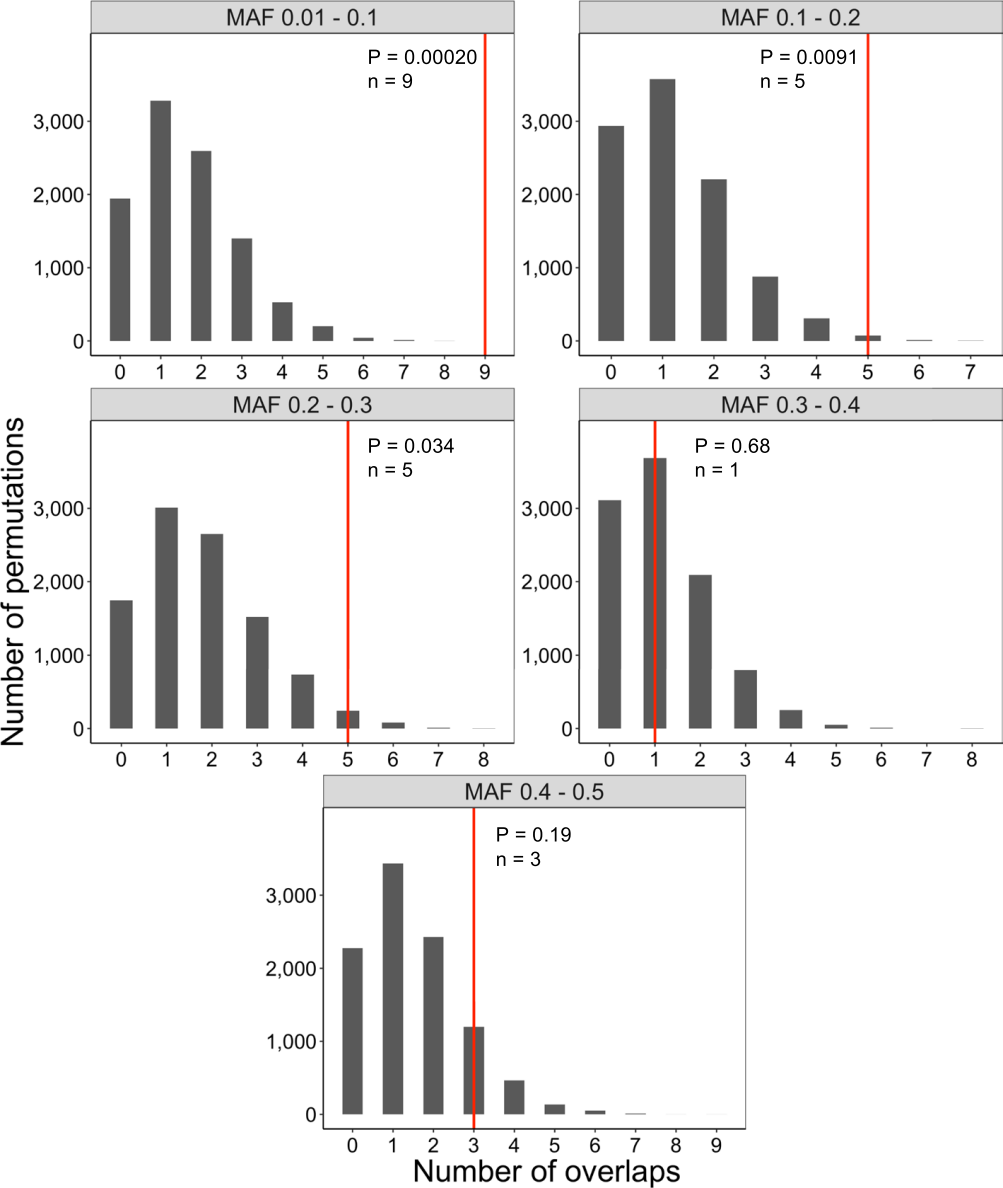
Enrichment of GWAS variants within smORFs. Permutation analysis testing enrichment of SNVs associated with disease/ other traits within smORFs segregated by MAF bins (facets) revealed a statistically significant enrichment among smORFs (Fisher’s method meta-analysis *P* = 4.96 × 10^–5^). *P* values were calculated by comparing the number of observed overlapping SNVs (red) to the number expected based on 10,000 permutations (grey histograms). Meta-analysis using Fisher’s method, H_0_: no significant enrichment of GWAS SNVs in smORFs. Abbreviation: MAF, minor allele frequency

We next explored the possible role of smORFs in human disease. We investigated whether common coding variants (MAF ≥ 0.01) in high-confidence smORFs showed evidence of association with human traits via a GWAS enrichment analysis. To do this, we identified common SNVs reported in the GWAS catalog that fell within coding regions of high-confidence smORFs [33]. We identified 25 SNVs overlapping smORFs with genome-wide significance (*P* < 5 × 10^–8^), including 18 non-synonymous and seven synonymous SNVs. These variants are implicated in a wide range of traits such as HDL cholesterol, HIV infection, breast cancer, and tuberculosis (Table S2). Next, we assessed whether the number of GWAS SNVs overlapping smORFs was greater than expected by chance. We compiled SNV lists from four SNV arrays commonly used in GWAS and identified all SNVs within ± 1 Mb that were in high linkage disequilibrium (r^2^ ≥ 0.8) of the genotyped variants on those four arrays [34, 35]. We then separated the SNVs into bins by minor allele frequency (MAF) and ran five separate permutation tests, one per MAF bin. Through the series of permutation tests, we observed a significant enrichment of trait-associated SNVs within our high-confidence set of smORFs (Fig. 4, *P* = 4.96 × 10^–5^, 10,000 permutations). We next explored the functional impact of the GWAS SNVs on the putative smORFs by computing Polyphen-2 scores ([36]. Similar to prior work in annotated genes [37], we identified no significant enrichment of nonsynonymous-damaging SNVs after performing a permutation analysis (N_damaging_ = 9, N_total_ = 18, Fisher’s exact test *P* = 0.36).

## Discussion

In this study, we utilized a comparative genomics approach to identify a set of high-confidence smORFs that are likely encoding functional micropeptides. We used datasets from two previously published studies that performed Ribo-seq to annotate novel smORFs [1, 4]. However, what remained unclear is the extent to which their predictions represented proteins with independent functions since ORFs can be translated as part of regulatory mechanisms or can be translational noise. Here, we provide further evidence of the functional significance of the predicted smORFs through analysis of constraint within human populations and evolutionary conservation. We compared the MOEUF and GERP score distributions of the predicted smORFs to annotated RefSeq genes, and after imposing strict thresholds, we generated a list of 3,326 high-confidence smORFs. High confidence smORFs tend to have similar distributions to less conserved genes, i.e., those with low and moderate pLI scores. We speculate this is because (1) smORFs may have a younger evolutionary age (younger genes tend to be shorter) and (2) lower statistical power to detect highly conserved genes [38–42]. Additionally, we assessed conservation of these high-confidence smORFs relative to the genome-wide background rate. By comparing the relative frequency of functional versus non-functional protein-coding variation, we showed that our high-confidence set is enriched with smORFs conserved at the protein-coding level, rather than merely being highly conserved non-coding regions. These results indicate that selective pressures are acting on the amino acid sequence of this set of high-confidence smORFs.

To explore the biological significance of our high-confidence smORFs, we asked whether genetic variation in these smORFs contributes to human traits and diseases. We showed that disease-associated SNVs with genome-wide significance are enriched in our high-confidence smORFs. In addition, we explored the functional impact of the GWAS SNVs on the smORFs by computing Polyphen-2 scores. Similar to prior work in annotated genes, we identified no significant enrichment of nonsynonymous-damaging variants among those implicated in human disease [37]. The reason for the lack of significant enrichment of damaging SNVs could be because (1) the GWAS SNVs used in our analysis are not the causal variants but rather are in linkage disequilibrium with causal SNVs, (2) Polyphen-2 predicted that the majority of smORF variants were damaging, limiting our statistical power, or (3) Polyphen-2 was developed for larger genes and might not be well-calibrated for smORFs. In this study, we analyze common variants (MAF ≥ 0.01), which have been the historical focus of GWAS. In the future, similar analyses can be applied to potential association results from large-scale whole genome sequencing efforts, which may include more rare variants [43, 44].

Finally, given that smORFs are commonly found in 5’ UTRs, 3’ UTRs, and introns of annotated genes, future work would need to leverage expression and proteomic molecular data to test if the GWAS associations with smORFs are mediated by SNVs that directly affect the smORF and/or nearby genes. A common follow-up analysis used to functionally evaluate GWAS signals is to perform statistical co-localization analysis. This analysis combines GWAS findings with functional data, such as expression quantitative trait loci, to examine if the same variant is causal in both [45, 46]. Currently, smORFs are not well-represented in most tissue and cell-specific expression datasets, such as the Genotype-Tissue Expression database [47], limiting the follow-up analyses we could perform to functionally interpret our GWAS findings.

To conclude, we present a robust workflow to identify high-confidence human smORFs using a comparative genetics approach. The alternate reading-frame analysis allowed us to assess conservation of the set of high-confidence smORFs at the protein-coding level and was critical to differentiating protein-coding regions from highly conserved non-coding regions. In addition, we found that smORFs are significantly enriched with disease-associated GWAS SNVs. Future studies analyzing novel human smORFs can follow our workflow (Fig. 1a), utilizing the empirical cutoffs for MOEUF and GERP scores as these are based on comparisons to known RefSeq genes. Since, our workflow leverages large population databases, we would only recommend our approach for analysis in other organisms where large whole-genome sequencing databases are available. Overall, our results are a step forward in showing that smORFs likely encode functional proteins implicated in human disease. Further advancements in experimental and computational tools are needed to improve annotation of novel smORFs and uncover their role in biology [48].

## Methods

### Dataset filtering

We downloaded datasets of predicted protein-coding ORFs from two previous published studies [1, 4], performed liftover to convert the datasets from hg19 to hg38 using the default parameters of UCSC Genome Browser’s web-interface liftOver tool, removed ORFs that were less than 10 codons and greater than 150 codons, and filtered to keep the longest coding DNA sequence for each predicted ORF [49]. For the Chen et al. dataset, we only considered smORFs that were labelled as upstream, new, start overlap, stop overlap, new isoform, downstream, and long out of frame [4]. Next, we intersected smORF exon regions with annotated RefSeq genes and the peptide atlas 'human Jan 2020' database, a database of peptides identified from tandem mass spectrometry experiments, using bedtools intersect [16, 17, 50]. We removed smORFs where the exon regions had a ≥ 50% overlap with exons of annotated RefSeq genes or vice versa. Additionally, smORFs where the exons regions had a 100% overlap with genomic coordinates of peptides from the Peptide Atlas database and ≥ 50% overlap with RefSeq genes were excluded. In addition to the initial filtering, we intersected the datasets to examine the similarity between the predictions from both studies. We performed two intersections, one to identify smORF predictions from both studies that were identical and the other considering a minimum of 1-basepair overlap.

### Constraint calculation

We adopted previous workflows to calculate MOEUF scores for known genes and novel smORFs [10]. We downloaded the gnomAD Hail table of all possible genomic variation with methylation and CpG context, filtered for regions in smORFs and RefSeq Select genes, performed liftover of Hail tables from hg19 to hg38, used ANNOVAR to annotate the "all possible variation" table with smORFs/gene function and gnomAD v3 WGS allele frequencies (based on 71,702 samples), identified all observed variants with minor allele frequency (MAF) < 0.001, used the mutation rate table to calculate expected variants based on 3-mer sequence context and finally calculated MOEUF scores for every smORF and RefSeq Select gene [16, 50–52]. Differences between this workflow and previously published pipelines to calculate MOEUF include using WGS data without coverage correction instead of whole-exome sequencing, ANNOVAR instead of Variant Effect Predictor, and obtaining the OEUF standard deviation from the combined set of all genes and smORFs. We validated this pipeline by comparing expected and observed variants for known genes to previously published scores and by calculating the correlation between expected and observed synonymous variants. We observed a high correlation between the number of expected missense variants per gene (*r* = 0.98) among 15,010 genes in both our results and the published score database. Consistent with prior correlations between the number of expected and observed synonymous variants per gene, we observed a high correlation (*r* = 0.98) across 30,334 genes and smORFs.

### Evolutionary conservation analysis

We extracted base-wise GERP scores for the list of smORFs from the GERP track on the UCSC Genome Table Browser [11, 53]. The average GERP score across smORF exons was calculated using bedtools intersect and bedtools group by [50].

### Damaging variant score

Polyphen-2 predicts the structural and functional impact of a missense substitution and assigns a score indicating the probability of the substitution being damaging. Scores range from 0 (benign) to 1 (damaging). Scores > 0.85 are more confidently predicted to be damaging and are termed ‘probably damaging’. smORF genetic variants were first functionally annotated using ANNOVAR’s gene-based annotation [52]. To compute the scores, the annotated variants were batch queried on a custom Polyphen-2 server [36].

### GWAS analysis and permutation testing

We filtered the NHGRI-EBI GWAS catalog, removing redundant single nucleotide polymorphisms (SNVs) and retaining only biallelic SNVs with a *p*-value < 5 × 10^–8^ (*n* = 57,099) [33, 52]. We downloaded all observed variants from gnomAD v3 database for every smORF and functionally annotated the variants using ANNOVAR [51, 52]. We then searched the annotated smORF variants against the GWAS catalog to identify SNVs associated with disease/traits. Next, we performed a permutation test to assess whether the enrichment of SNVs identified by GWAS in smORFs was significant. We compiled SNV lists from four SNV arrays (Affymetrix SNV 6.0, Illumina Human1M-Duo, Illumina HumanOmni1_Quad, and Illumina HumanHap550), accessed through the SNV/CNV track on UCSC Genome Table Browser [53], performed liftover to convert these from hg19 to hg38, removed redundant SNVs and filtered to keep biallelic SNVs (*n* = 1,569,244). Using plink1.9 software, we identified all SNVs that were in linkage disequilibrium (r^2^ ≥ 0.8) and within 1 Mb of the SNVs in the SNV lists [34, 35]. We then separated the compiled SNV list into bins by MAF (1–10%, 10–20%, 20–30%, 30–40% and 40–50%) and ran five separate permutation tests, one per MAF bin. We sampled variants with replacement from each SNV list, corresponding to the number of variants from the GWAS catalog in each MAF bin, and calculated the number of SNVs overlapping smORF exons. For each test, we computed the *p*-value based on 10,000 permutations and the observed number of SNVs identified by GWAS overlapping smORFs. We computed the meta-analysis *p*-value using Fisher's method, which combines *p*-values from independent tests into a single test statistic.

In addition, we performed a permutation analysis testing whether there was an enrichment of SNVs scored as damaging by Polyphen-2 amongst the GWAS SNV hits overlapping our high-confidence smORFs. We sampled variants with replacement from a set of Polyphen-2 scored nonsynonymous smORF SNVs (MAF 1–50%) and computed the *p*-value based on 10,000 permutations and the observed number of GWAS SNVs scored as damaging.

## Supplementary Information

**Additional file 1:** **Table S1. **List of high-confidence smORFs and known smORFs.

**Additional file 2:** **Table S2. **List of smORFs carrying disease-associated SNVs from GWAS catalog.

## Data Availability

The Martinez et al. [1] dataset analyzed in this study is included in the supplementary information (supplementary data 1) of this published article, Martinez TF, Chu Q, Donaldson C, Tan D, Shokhirev MN, Saghatelian A: Accurate annotation of human protein-coding small open reading frames. Nat Chem Biol 2020, 16(4):458–468. The Chen et al*.* [4] dataset analyzed in this study is available in the GEO database, https://www.ncbi.nlm.nih.gov/geo/query/acc.cgi?acc=GSE131650 (GSE131650_orfratings.bed.gz). Filtered datasets are within the manuscript and its supporting information files.
